# Soil Moisture Data Assimilation to Estimate Irrigation Water Use

**DOI:** 10.1029/2019MS001797

**Published:** 2019-11-17

**Authors:** R. Abolafia‐Rosenzweig, B. Livneh, E.E. Small, S.V. Kumar

**Affiliations:** ^1^ Department of Civil, Environmental, and Architectural Engineering University of Colorado Boulder Boulder CO USA; ^2^ Cooperative Institute for Research in Environmental Science (CIRES) University of Colorado Boulder Boulder CO USA; ^3^ Geological Sciences University of Colorado Boulder Boulder CO USA; ^4^ Hydrological Sciences Laboratory, NASA Goddard Space Flight Center Greenbelt MD USA

**Keywords:** irrigation, data assimilation, soil moisture, particle batch smoother, remote sensing, land surface model

## Abstract

Knowledge of irrigation is essential to support food security, manage depleting water resources, and comprehensively understand the global water and energy cycles. Despite the importance of understanding irrigation, little consistent information exists on the amount of water that is applied for irrigation. In this study, we develop and evaluate a new method to predict daily to seasonal irrigation magnitude using a particle batch smoother data assimilation approach, where land surface model soil moisture is applied in different configurations to understand how characteristics of remotely sensed soil moisture may impact the performance of the method. The study employs a suite of synthetic data assimilation experiments, allowing for systematic diagnosis of known error sources. Assimilation of daily synthetic soil moisture observations with zero noise produces irrigation estimates with a seasonal bias of 0.66% and a correlation of 0.95 relative to a known truth irrigation. When synthetic observations were subjected to an irregular overpass interval and random noise similar to the Soil Moisture Active Passive satellite (0.04 cm^3^ cm^−3^), irrigation estimates produced a median seasonal bias of <1% and a correlation of 0.69. When systematic biases commensurate with those between NLDAS‐2 land surface models and Soil Moisture Active Passive are imposed, irrigation estimates show larger biases. In this application, the particle batch smoother outperformed the particle filter. The presented framework has the potential to provide new information into irrigation magnitude over spatially continuous domains, yet its broad applicability is contingent upon identifying new method(s) of determining irrigation schedule and correcting biases between observed and simulated soil moisture, as these errors markedly degraded performance.

## Key Points

- We present a framework to estimate irrigation from soil moisture data assimilation using a particle batch smoother
- A suite of synthetic experiments is conducted to systematically evaluate the impacts of known error sources
- The method is most sensitive to unknown irrigation timing within a day and systematic errors between observed and modeled soil moisture

## 1 Introduction

Irrigated land produces more than 40% of global food and agricultural commodity outputs on only 20% of agricultural land worldwide (Vörösmarty & Sahagian, 2000). Irrigation is the largest anthropogenic use of fresh water, consuming about 70–75% of the world's freshwater (Zhang et al., 2017), directly contributes to groundwater depletion (Famiglietti et al., 2011; Rodell et al., 2009; Scanlon et al., 2012) and impacts the water and energy cycles (Haddeland et al., 2006; Jiang et al., 2014; Ozdogan et al., 2010), underscoring the importance of quantifying the magnitude of this flux. Despite its importance, few methodologies exist to produce a continuous, observationally based irrigation estimate. Most existing irrigation data sets focus on mapping the occurrence of irrigation (Deines et al., 2017; Ozdogan & Gutman, 2008; Salmon et al., 2015) or rely solely on models to estimate irrigation magnitude (Haddeland et al., 2006; Jiang et al., 2014; Ozdogan et al., 2010). As it stands there exist few published methodologies designed to estimate irrigation magnitude suitable for global application. Here, we present a new methodology to use data assimilation (DA) with land surface model (LSM) simulated soil moisture (SM) to estimate daily to seasonal irrigation magnitude at the model's spatial resolution.

Historically, irrigation water use has been monitored using a power consumption coefficient assumption, which estimates the amount of water being pumped for irrigation as a function of the power an irrigation well draws (Hurr & Litke, 1989). However, the relatively small number of these in situ irrigation observations limits the large‐scale applicability of this method (Brocca et al., 2018). Most large‐scale irrigation data sets rely on statistical surveys or simply identify areas equipped for irrigation (Siebert and Döll, 2000; Thenkabail et al., 2005; Salmon et al., 2015). While these maps are generally spatially consistent over commonly irrigated areas, issues of accuracy arise at larger scales that could be improved through the incorporation of remote sensing (Liu et al., 2018). Attempts to map irrigation extent with remote sensing have leveraged vegetation indices from Advanced Very High Resolution Radiometer (Loveland et al., 2000; Thenkabail et al., 2008), Moderate Resolution Imaging Spectroradiometer 250 (Ambika et al., 2016; Ozdogan & Gutman, 2008; Teluguntla et al., 2017), and Landsat 30 (Deines et al., 2017; Ozdogan et al., 2006; Pun et al., 2017) satellite products. Most recently, remotely sensed SM from Sentinel‐1 has shown potential to compliment vegetation index irrigation mapping techniques to produce irrigation maps at high spatial resolutions and relatively high temporal resolutions (Bousbih et al., 2018; Gao et al., 2018).

LSMs have served a unique role in irrigation mapping. While they have traditionally lacked a formal irrigation scheme, it is possible to infer irrigation by contrasting simulated land surface evapotranspiration with remotely sensed observations that implicitly include an irrigation signal (Romaguera et al., 2012). Over the past two decades, efforts to improve modeled representation of irrigation have sought to assess the effects of irrigation on LSM‐derived water and energy balances and to improve the representation of managed lands in land surface schemes. LSM studies have shown that irrigation increases SM leading to greater evapotranspiration with increases in latent heat flux and decreases in both sensible heat flux and coupling between SM and latent heat flux in water limited environments (Badger & Dirmeyer, 2015; de Rosnay, 2003; Haddeland et al., 2006; Jiang et al., 2014; Lawston et al., 2015; Mahmood & Hubbard, 2002; Ozdogan et al., 2010; Tang et al., 2007). This repartitioning of the surface energy and water balances causes lower surface air temperature and elevated atmospheric water vapor that contributes to the greenhouse effect (Boucher et al., 2004; Haddeland et al., 2006; Jiang et al., 2014; Lawston et al., 2015; Ozdogan et al., 2010; Tang et al., 2007). Frameworks to model irrigation within LSMs follow simple rules based on balancing available water supply with plant and atmospheric water demand (de Rosnay, 2003; Haddeland et al., 2006; Ozdogan et al., 2010; Tang et al., 2007). However, uncertainties in irrigation mapping and weather data can result in variations of irrigation water demand of about 30% (Wisser et al., 2008). Irrigation estimates have typically been validated with local reports of annual consumptive water use (Haddeland et al., 2006; Ozdogan et al., 2010) or evapotranspiration as a proxy for irrigation given the lack of irrigation monitoring (Lawston et al., 2015). Therefore, despite recent advances, model irrigation studies remain largely under‐validated, particularly at time scales less than 1 month.

Given the importance of constraining and validating these irrigation studies and lack of in situ data, attempts to use remote sensing to monitor agricultural water use have been explored, primarily using remotely sensed evapotranspiration (Droogers et al., 2010; Sun et al., 2017; van Dijk et al., 2018; Wu et al., 2015) and SM (Brocca et al., 2018; Zaussinger et al., 2018). While SM retrievals are now available from a number of passive microwave and scatterometer‐based instruments (El Hajj et al., 2017; Entekhabi et al., 2010; Gao et al., 2017; Kerr et al., 2012; Kim et al., 2015; Wagner et al., 2013), a key challenge lies in whether remotely sensed SM can adequately capture irrigation signals. Recent studies have concluded that the Soil Moisture Active Passive (SMAP) satellite, Sentinel‐1 satellites, and the Advanced Scatterometer can reliably detect irrigation signal, and the Soil Moisture Ocean Salinity mission, Advanced Microwave Scanning Radiometer for the Earth Observing System, the Advanced Microwave Scanning Radiometer 2, and the European Space Agency Climate Change Initiative soil moisture product can detect irrigation signal but with lower skill (Bousbih et al., 2018; Escorihuela & Quintana‐Seguí, 2016; Gao et al., 2018; Jalilvand et al., 2019; Kumar et al., 2015; Lawston et al., 2017; Zhang et al., 2018). Recently, Brocca et al. (2018) input remotely sensed SM into an inverted soil water balance equation to calculate monthly irrigation amounts during nonrainy satellite overpasses. Jalilvand et al. (2019) built on Brocca et al. (2018) by removing bias from estimated irrigation by estimating model bias over nonirrigated or rain‐fed cropland areas and used these biases for correcting the simulation at irrigated pixels. Zaussinger et al. (2018) quantified seasonal irrigation by attributing biases between remotely sensed soil wetting and modeled soil wetting to irrigation. Although remotely sensed SM captures irrigation signals, these retrievals alone are insufficient to assess spatiotemporally continuous estimates of irrigation and its effects on the water and energy cycles. Studies like Lievens et al. (2017) that assimilate observations from both SMAP and Sentinel‐1 leverage the strengths of each and have the potential to ameliorate issues from prior studies that relied exclusively on SMAP retrievals to estimate irrigation magnitude given that the coarse spatial resolution failed to resolve local irrigation practices (Brocca et al., 2018; Zaussinger et al., 2018). Sentinel‐1 SM observations (10 m resolution) may be more appropriate than SMAP observations (3–36 km) to resolve local irrigation practices in many regions worldwide where the footprint of irrigation application is smaller than the SMAP resolution. However, a key limitation of Sentinel‐1 retrievals are their less frequent overpass intervals (6 days) (Bousbih et al., 2018; Gao et al., 2018).

The goal of DA is to leverage the strengths of spatiotemporally continuous model simulations, for example, constrained water and energy balances, with the veracity of observations, using observations to “correct” key model states such as SM (Lievens et al., 2015; Reichle et al., 2008). Correction of model states with DA has been used to provide more accurate estimates of model outputs such as SM, streamflow, and snow water equivalent (Dong et al., 2015; Lievens et al., 2015; Margulis et al., 2015; Smyth et al., 2019) and correct model inputs, such as precipitation (Crow et al., 2011; Crow & Bolten, 2007; Crow & Ryu, 2009; Zhan et al., 2015). A key assumption in most DA techniques is that the errors in observations and model forecasts are strictly random and that on average, the observations and model estimates agree with true Earth states. In reality, biases are unavoidable, and it is difficult to attribute the bias to the model or observations (Kumar et al., 2012). Often these biases are treated prior to assimilation through cumulative distribution function (CDF) matching to essentially rescale observations to the modeled climatology.

However, a critical problem arises in CDF‐matching observations to a model climatology, in particular when the model physics do not account for processes such as irrigation. The goal of CDF‐matching is to map the observed climatology to the land model, which intends to erase biases between the land model and observations due to instrument and retrieval errors. Yet, when the LSM does not account for irrigation, the CDF rescaling also removes the impact of unmodeled processes, such that observed irrigation signal also gets erased (Kumar et al., 2015). Thus, removing biases between observations and the model is important in DA, and treatment of biases arising from unmodeled processes (i.e., irrigation) represents an unresolved challenge. Ongoing research exploring DA over irrigated regions to resolve or circumvent this issue includes calibrating LSMs in NASA's Land Information System to in situ SM observations, CDF‐matching observations to the climatology of an LSM using an irrigation scheme, and assimilating multiple remotely sensed variables that contain irrigation signal, for example, evapotranspiration or evaporative stress index. Because bias correction over irrigated land remains an unresolved issue, this study follows Dee (2005) and uses a bias‐blind DA approach without any a priori bias correction. Biases are documented, and their impact is evaluated across the conterminous U.S. (CONUS). The inferences from this study are expected to contribute toward the development of bias correction strategies that preserves signal of unmodeled processes.

Here, we present a methodology that uses SM DA to estimate irrigation magnitude and improve understanding of irrigation's effects on surface SM. We apply and evaluate the methodology using a suite of synthetic DA experiments (Kumar et al., 2012; Kumar et al., 2015; Reichle et al., 2008) that use SM outputs from a control simulation as a surrogate for remotely sensed SM retrievals. While we do not directly assimilate remotely sensed SM, we impose categorical errors in the experiments using the characteristics of SM from NASA's SMAP satellite as a way to systematically evaluate both the performance and limitations of the method in an applied context. Evaluations are presented in the context of SMAP retrievals because these have been shown to yield the most accurate SM estimates relative to other sensors (Chen et al., 2018; Kumar et al., 2018; Lievens et al., 2017), although the method can be applied to any SM product or land model. This study follows an approach similar to Crow et al. (2011), except that unlike Crow et al. (2011) who were interested in improving estimates of precipitation, here we seek to quantify water input from irrigation. This manuscript is organized around a suite of synthetic experiments, presented to systematically evaluate the impacts of known, SMAP‐based error sources on the DA system. We seek to evaluate the impact of the following system characteristics on the performance of estimated irrigation: (i) the window length of the DA smoothing algorithm, (ii) the frequency of satellite overpasses, (iii) noise in the SM data, (iv) relative magnitude of irrigation compared to precipitation, (v) biases between the LSM and the satellites, and (vi) the challenge of unknown irrigation timing.

## 2 Materials and Methods

### 2.1 Approach

We use the particle batch smoother (PBS) DA method (Dong et al., 2015; Margulis et al., 2015; Vrugt et al., 2013) to estimate the unmodeled irrigation process on the basis of minimizing errors between simulated and observed SM states. Particle‐type DA algorithms have been used to successfully increase accuracy of moisture states and fluxes in small‐scale problems assimilating in situ SM observations (Dong et al., 2015) and in large‐scale problems assimilating remotely sensed SM observations (Crow et al., 2011; Crow & Ryu, 2009). An important distinction of the PBS is that it tracks the accuracy of individual model simulations, for example, particles, and gives more weight to accurate particles. In contrast, the other common assimilation method—the Ensemble Kalman Smoother (EnKS)—adjusts, for example, nudges, the state of the model closer to the observed state estimate. We elect to use the PBS over the EnKS because estimating model inputs, that is, precipitation and irrigation, from the EnKS requires parameters external to the land model that are difficult to calibrate (Crow et al., 2011; Crow & Ryu, 2009). Conversely, particle accuracy, or weights from the PBS, can be directly translated into respective particle forcings to determine the best estimate of water input (the sum of irrigation and precipitation), from which known precipitation can be subtracted to estimate irrigation. The implicit assumption is that accurate SM states are the product of accurate model forcing. Application of a known amount of irrigation in these experiments allows us to comprehensively validate the method under key sources of error introduced sequentially (i.e., one at a time) and in combination. We elect to specify irrigation amount, rather than use observations or census data, so as to avoid confounding the analysis with sparse and biased data (Brocca et al., 2018; Kumar et al., 2015). Because the objective of this manuscript is to critically evaluate a new approach, we elect to specify a “truth” irrigation signal which allows for the systematic diagnosis of the known error sources as well as overcome data limitations (Brocca et al., 2018), both of which are needed for a comprehensive validation. Hence, all DA experiments in this study use synthetic observations derived from LSM simulations rather than remotely sensed retrievals.

Core experimentation is conducted on a single grid cell in a heavily irrigated region of Nebraska, with an extended analysis performed on other irrigated regions across CONUS to evaluate the role of climate on method performance. Synthetic experiments assume perfect knowledge of when and where irrigation is present. Although irrigation maps accurately predict the location of irrigation (Ambika et al., 2016; Bousbih et al., 2018; Gao et al., 2018; Ozdogan & Gutman, 2008; Teluguntla et al., 2017), their temporal resolution is not fine enough to determine temporal boundaries of the irrigated season. In this study, we assume the irrigation season follows Yonts (2002) and acknowledge that nonsynthetic applications of this method will require identification of the irrigation season at the model's spatial.

**Figure 1:**
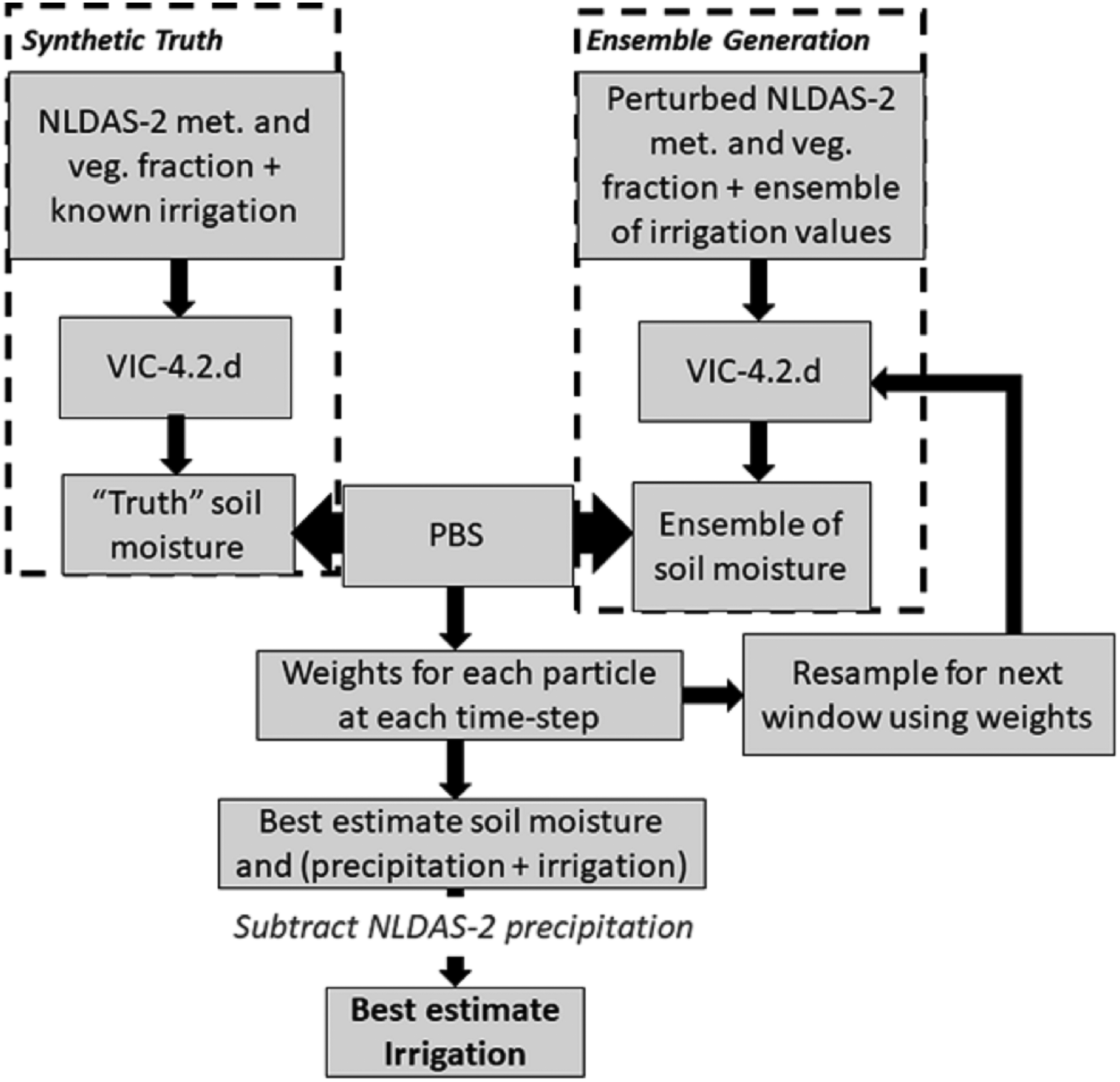
Structure of synthetic data assimilation experiment.

**Figure 2:**
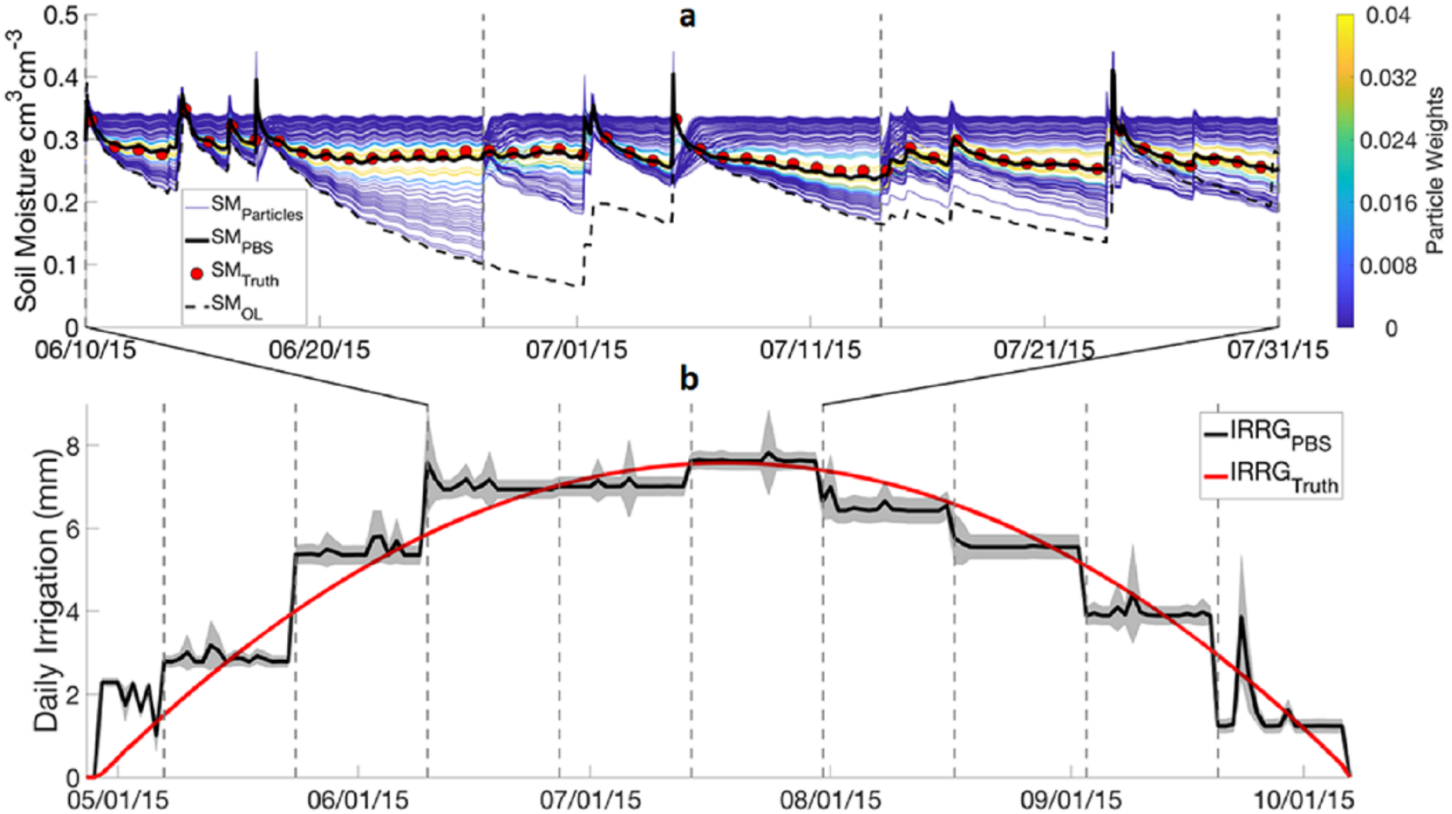
(a) Time series for subsection of the irrigation season of 99 particles colored by weight assigned based on proximity to the *SM*_*truth*_ shown as red circles, with the weighted particle average plotted as a solid black line and OL SM without irrigation plotted as a dotted black line. (b) The corresponding time series of *IRRG*_*PBS*_ (black line), *σ*_*IRRG* − *PBS*_ (gray shading), and *IRRG*_*truth*_ (red line).

The suite of synthetic DA experiments uses an identical twin experiment set up (Kumar et al., 2015), presented in Figure 1. All simulations are run over a single model grid cell in Nebraska. A 2‐year spin‐up generated initial conditions for the DA experiments. The 2015 irrigation season (29 April to 6 August) (Yonts, 2002) is evaluated. The variable infiltration capacity (VIC) LSM simulation forced with the NLDAS‐2 data is termed the open loop (OL) integration (Table 1). The VIC LSM simulation forced with NLDAS‐2 data and a prescribed quantity of irrigation is used as the “truth” simulation (Table 1). From the truth simulation, observations are generated. These synthetic observations are assimilated with an ensemble of particles forced with NLDAS‐2 data across a range of irrigation magnitudes. Each particle receives weights from the PBS over defined fixed window intervals. Weights are traced to particle forcings to describe a posterior probability density function (PDF) of precipitation plus irrigation, while also managing the population of particles. The population is managed by resampling from a set of preferential, that is, high‐weighted, particle states at the beginning of each new window using the sequential importance resampling methodology (Gordon et al., 1993; Weerts & El Serafy, 2006), and particles with lower weights are generally discontinued (Figure 2a). The expected value of the posterior PDF yields the single best estimate of precipitation plus irrigation for each smoothing window. NLDAS‐2 precipitation is subtracted from this best estimate of precipitation plus irrigation to yield the single best estimate of irrigation for each smoothing window. Here, the standard deviation of irrigation's posterior PDF is used to represent the uncertainty of the estimated irrigation (Figure 2b).

**Table 1:** Definitions of Key Variables and Terms Used in Synthetic Data Assimilation Experiments

Variable/Term	Definition
*P*_*Obs*_	Gridded historical precipitation
*IRRG*_*truth*_	Synthetic irrigation following published weekly water use patterns in Western Nebraska (Yonts, 2002)
*A*_*truth*_	Aggregate of observed precipitation and truth irrigation, *A*_*truth*_ = *P*_*OBS*_ + *IRRG*_*truth*_. Used as forcing in the “Truth Simulation”
Open loop simulation	Simulation designed to portray nonirrigated land
Truth simulation	Simulation designed to portray irrigated land
*SM*_*OL*_	Surface SM outputs from open loop simulation
*SM*_*truth*_	6 a.m. surface SM outputs from truth simulation. Used as synthetic observations in DA experiments
*SM*_*truth + Overpass*_	6 a.m. surface SM outputs of truth simulation on days of valid SMAP overpasses. Used as synthetic observations in DA experiments
Particles	Simulations forced with *P*_*Obs*_ + precipitation perturbations. Precipitation perturbations account for noise in *P*_*Obs*_ and unknown irrigation quantities
*P*_*particle*_	Precipitation used to force particles in PBS simulations
*A*_*PBS*_	Best estimate precipitation + irrigation from particle batch smoother algorithm
*IRRG*_*PBS*_	Best estimate irrigation from particle batch smoother algorithm. (*A*_*PBS*_–*P*_*Obs*_)
*σ*_*IRRG* − *PBS*_	Standard deviation of the discrete posterior PDF of the irrigation ensemble, used here as a measure of *IRRG*_*PBS*_ uncertainty

*Note*. Additional details can be found in section 3 describing data sources.

Key variables are defined in section 2.1.1, and the basic implementation of the PBS is described in section 2.1.2. The experimentation on methodological error sources follows for window length (section 2.2.1), frequency of observations (section 2.2.2), observational noise (section 2.2.3), irrigation magnitude (section 2.2.4), model bias (section 2.2.5), irrigation application timing (section 2.2.6), and a comprehensive evaluation combining frequency of observations, observational noise, and irrigation application timing (section 2.2.7), with the metrics for performance evaluation presented in section 2.3.

#### 2.1.1 Definitions

Key variables used in the synthetic DA experiments are referenced in Table 1. *IRRG*_*truth*_ is created from a spline interpolation of weekly corn water use (Yonts, 2002). The aggregate of both sources of water, *A*_*truth*_ = *P*_*OBS*_ + *IRRG*_*truth*_, is used to force the synthetic truth LSM simulation. Irrigation is added to the LSM as supplemental precipitation forcing. For consistency with the SMAP 6 a.m. overpass timing (Entekhabi et al., 2014; Jackson et al., 2012), LSM outputs at 6 a.m. local time are used as the truth synthetic observations, *SM*_*truth*_, in the PBS algorithm.

#### 2.1.2 Precipitation Perturbations and the Particle Batch Smoother

Here we describe how the PBS algorithm is implemented. We refer the reader to Dong et al. (2015) for a comprehensive and general presentation of the PBS.

Precipitation is perturbed over all time steps to generate the suite of 99 particles by introducing multiplicative Gaussian noise with a 10% standard deviation, *N*(0, 0.1), accounting for random noise in precipitation observations. Here, *ε* is a Monte Carlo sample from this distribution. A second perturbation, *IRRG*(*r*), is superimposed during the irrigated season. Precipitation perturbations (*η* ^*i*^) and precipitation forcing applied to particle simulations (*P* ^*i*^_*particle*_) are described by equations (1a) and (1b), respectively.

(1a) $$\eta^{i}=P_{\mathrm{obs}*}\;\varepsilon^{i}+{\text{IRRG}}^{i}\left(r\right)$$

(1b) $${P^{i}}_{\text{particle}}=P_{\mathrm{obs}}+\eta^{i}$$

where *η* ^*i*^ is the perturbation added to *P*_*obs*_, and *IRRG* ^*i*^(*r*) is a random sample from a uniform distribution range, *r*, of irrigation magnitudes, and *i* denotes the *i*th particle in the ensemble, hence *P*_*particle*_ is a 99x1 vector at each time step. The range of superimposed irrigation perturbations, *r*, is 0–30 mm day^−1^ during the irrigation season and *r* = 0 elsewhere. A uniform distribution is used because we assume no prior knowledge of irrigation magnitude, thus the entire possible range of irrigation is considered equally. *IRRG* ^*i*^(*r*) is applied continuously each day to the *i*th particle during the irrigation season, matching the same timing of *IRRG*_*truth*_. We apply irrigation continuously each day given that 80% of irrigated lands in Nebraska rely on sprinkler systems (Johnson et al., 2011) that are commonly in use 22 hr per day (Ross, 1997). Multiple irrigated fields are observed by each 9 km SMAP sensing pixel, so we assume irrigation is applied continuously in each coarse pixel during a growing season.

An ensemble of model states evolves in parallel using the forward model:

(2) $${x^{i}}_{t}=f\;\left({x^{i}}_{t-1},{u^{i}}_{t},b^{i}\right)+{w^{i}}_{t}$$

where *x* ^*i*^_*t*_ is the model state (SM) of the *i*th particle at time *t*, *u* ^*i*^_*t*_ are the perturbed forcing data, *b* ^*i*^ is a vector of time invariant model parameters, *w* ^*i*^_*t*_ is the model error, and *f* is the forward model (VIC). Here, *x* ^*i*^_*t*_ is a 99x1 vector because SM is the only assimilated state.

Posterior expected values for precipitation plus irrigation, *A*_*PBS*_, are calculated as the mean precipitation forcing of the discrete posterior density given from the PBS. The posterior density is fully described by the conditional PDF given by Bayes' theorem (Margulis et al., 2015):

(3) $$p\left(y_{t-L+1:t}\;|\;x_{t-L+1:t}^{i}\right)=\prod_{j=t-L+1}^{t}\;\frac{1}{{\left(2\pi \right)}^{n/2}\det{\left(C_{V}\right)}^{1/2}}e^{\left(-0.5{\left(y_{j}-x_{j}^{i}\right)}^{T}C_{V}^{-1}\left(y_{j}-x_{j}^{i}\right)\right)}$$

or the likelihood of observed states, *y*, given particle estimates, *x*, in window *t* − *L +* 1:*t*, where *L* represents the length of the window. In a special case where *L* = 1, observations are assimilated sequentially, that is, the particle filter. In cases where *L* > 1, the PBS assimilates observations within a window in a single batch. The likelihood function is specified as a Gaussian PDF that is a function of observation error covariance (*C*_*V*_), residuals between simulated SM and all observations within a window, and the number of assimilated states or fluxes (*n*). In this case, *n* = 1 because only SM is assimilated. Particles that produce SM states with small residuals relative to observed states receive higher likelihood estimates compared to particles that produce SM states with larger residuals. *C*_*V*_ plays a large roll in controlling the spread of weights in that a smaller *C*_*V*_ results in fewer particles with high weights. Synthetic experiments that use *SM*_*truth*_ as observations assume *C*_*V*_ to be unknown, and the model's ensemble variance at the time of assimilation is used as a proxy for *C*_*V*_. A weakness of this assumption is that observation uncertainty becomes a function of window length, since the ensemble spread increases for longer window lengths. Experiments that use these perfect observations provide an exposition of the method, but in our core experimentation we use more realistic values of prescribed observational error, where this prescribed error is used for *C*_*V*_. We advise the reader to use prescribed product errors for *C*_*V*_ in the likelihood calculation for any application of this method.

Weights for each particle are equated to likelihood estimates. Weights defining the discrete posterior PDF are normalized between 0 and 1 of likelihood estimates:

(4) $$w_{t}^{i}=\frac{p\left(y_{t-L+1:t}\;|\;x_{t-L+1:t}^{i}\right)}{\sum_{i=1}^{N}p\left(y_{t-L+1:t}\;|\;x_{t-L+1:t}^{i}\right)}$$

where *w* ^*i*^_*t*_ is the normalized weight assigned to the entire window and is a 99x1 vector at each time step, which is repeated at each time step in a fixed window; that is, weights are constant within a fixed window.

We use sequential importance resampling (Gordon et al., 1993; Weerts & El Serafy, 2006) to avoid degeneracy (collapse) of the posterior weights after several updates (Margulis et al., 2015). The resampling process is analogous to rolling an *N*‐sided loaded die, where *N* is the number of particles generated; here *N* = 99. The probability of rolling each side of the die is defined by weights calculated in equation (4). The die is rolled *N* times. Each time the die lands on a particle's side, a new particle is generated from the particle's state at the end of the previous fixed window and propagated to the start of the current fixed window. Hence, particles with higher weights have higher probabilities of propagating their states to be initial conditions in the subsequent window.

*A*_*PBS*_, the expected time series of precipitation plus irrigation, is calculated as

(5) $$A_{\mathrm{PBS},t}=\sum_{i=1}^{N}w_{t}^{i}\;P_{\text{particle},t}^{i}$$

where *P* ^*i*^_*particle*,*t*_ is the precipitation forcing particle *i* at time *t*. The estimated irrigation from PBS simulations, *IRRG*_*PBS*_, is calculated by subtracting *P*_*Obs*_ from *A*_*PBS*_.

We quantify the uncertainty of *IRRG*_*PBS*_, as the standard deviation of the discrete posterior PDF of the irrigation ensemble (Montgomery & Runger, 2013):

(6) $$\sigma_{\text{IRRG}-\mathrm{PBS}}=\sqrt{\sum_{i=1}^{N}w_{t}^{i}\;{\left({\text{IRRG}}^{i}\left(r\right)-{\text{IRRG}}_{\mathrm{PBS}}\right)}^{2}\;}$$

### 2.2 Sensitivity Experiments of the DA System

The performance of the DA system is evaluated against the error sources described below and presented in Table 2. DA performance has been shown to be sensitive to initial conditions in experiments assessing different observational frequency and window length (Dong et al., 2015). For this reason, the first two experiments were run at least 10 times with different initial conditions, for example, different starting dates, as a way to comprehensively evaluate model performance. Initial conditions were taken from a 2‐year spin‐up simulation that used identical inputs as the truth simulation excluding irrigation forcing. Experiments that include random observation noise (sections 2.2.3 and 4.3; sections 2.2.4 and 4.4; and sections 2.2.7 and 4.7) were run 20 times applying a unique time series of random noise to account for multiple realizations of random noise, since these results were shown to be particularly sensitive to different instances of random noise.

**Table 2:** Experiment Descriptions With Corresponding Methods and Results Sections

Experiment name	Relevant methods and results sections	Experiment description
Window length	2.2.1 and 4.1	Evaluate the impact of 1‐ to 30‐day windows on irrigation performance. Assimilate daily *SM*_*truth*_ with zero noise or bias. Force particle and truth simulations with irrigation applied on a continuous schedule.
Frequency of observations	2.2.2 and 4.2	Evaluate the impact of hypothetical satellite overpass intervals of 1 to 9 days using a short, medium, and long window length, 10, 16, and 24 days, respectively. Assimilate *SM*_*truth*_ with zero noise or bias. Force particle and truth simulations with irrigation applied on a continuous schedule.
Observation noise	2.2.3 and 4.3	Evaluate irrigation performance when synthetic observations are imposed with 0‐mean Gaussian noise with standard errors of 0.01, 0.02, 0.03, 0.04, and 0.05 cm^3^ cm^−3^ using a short, medium, and long window length, 10, 16, and 24 days, respectively. Assimilate *SM*_*truth + Overpass*_ with zero bias. Force particle and truth simulations with irrigation applied on a continuous schedule.
Irrigation magnitude	2.2.4 and 4.4	Evaluate irrigation performance across a range of irrigation/precipitation ratios using a short, medium, and long window length, 10, 16, and 24 days, respectively. Force truth simulations with varying combinations of *P*_*Obs*_ and *IRRG*_*truth*_, rescaling *P*_*Obs*_ and *IRRG*_*truth*_ via scalar multipliers to maintain the same magnitude of *P*_*Obs*_ + *IRRG*_*truth*_ for all experiments while varying the ratio of *IRRG*_*truth*_/*P*_*Obs*_. Conduct experiments for each truth simulation. Assimilate *SM*_*truth + Overpass*_ imposed with 0‐mean Gaussian noise with a standard error of 0.03 cm^3^ cm^−3^ and zero bias. Force particle and truth simulations with irrigation applied on a continuous schedule.
Model‐observation bias	2.2.5 and 4.5	Evaluate the impact of systematic bias between the model particles and truth simulation using a medium window length, 16 days. Assimilate *SM*_*truth + Overpass*_ imposed with static systematic biases (−0.2 to 0.2 cm^3^ cm^−3^) and zero random noise. Force particle and truth simulations with irrigation applied on a continuous schedule.
Irrigation application timing	2.2.6 and 4.6	Evaluate the impact of unknown irrigation timing and discontinuous irrigation schedules on irrigation performance using a long, 24 days, window length. Five experiments are conducted with different combinations of truth irrigation timing and particle irrigation timing. Force the first truth simulation with irrigation applied continuously (as done previously). Force the second truth simulation with irrigation applied every day from 4 a.m.–10 a.m. Force the third truth simulation with irrigation applied all day, 2 days per week. In the first three experiments, the particle simulations are forced with irrigation applied on a continuous schedule, which assumes no a priori knowledge of irrigation timing. Force the fourth truth simulation with irrigation applied all day, 2 days per week. Force the fifth truth simulation with irrigation applied every day from 4 a.m.–10 a.m. In the fourth and fifth experiments, particle simulations are forced with irrigation applied on the same schedule as truth irrigation to investigate the impact of a priori knowledge of irrigation timing. Daily *SM*_*truth*_ is assimilated with zero noise or bias for all five experiments.
Comprehensive evaluation	2.2.7 and 4.6	Evaluate the impact of unknown irrigation timing, discontinuous irrigation schedules, irregular overpass intervals, and observational noise on irrigation performance. Conduct five irrigation timing experiments explained in section 2.2.6 when synthetic observations are imposed with 0‐mean Gaussian noise with a standard error of 0.04 cm^3^ cm^−3^. Assimilate *SM*_*truth + Overpass*_ with zero bias.

#### 2.2.1 Window Length

Window length is a potentially limiting factor for the performance of the presented method given that weights are constant from each window. This has the effect of producing constant irrigation estimates during each window, assuming no random noise from precipitation, that is, *ε* in equation (1a) is 0. However, in the presented synthetic DA experiments, we assume random noise in precipitation observations exist, so discrepancies between *P*_*particle*_ and *P*_*Obs*_ exist due to both random perturbations and irrigation perturbations (equation (1a)) causing irrigation estimates in each fixed window to not be constant (Figure 2b). A trade‐off exists in using shorter versus longer window lengths, since irrigation estimates using shorter window lengths can capture finer temporal variations in truth irrigation although noisy observations can be potentially impactful. Longer window lengths miss high‐frequency fluctuations, but since they include more observations, they produce more stable irrigation estimates that are less sensitive to noisy observations than shorter windows. Here, synthetic DA experiments are conducted where daily *SM*_*truth*_ is assimilated with particles using window lengths of 1–30 days. We assess the median statistics, or measure of the central tendency, of each window. The particle filter is considered a special case of the PBS when the window length is 1 day. Understanding that trade‐offs between shorter and longer window lengths are present, we evaluate the method's sensitivity in the context of three example windows, 10, 16, and 24 days, in latter experiments.

#### 2.2.2 Frequency of Observations

Remotely sensed surface SM retrievals generally occur at infrequent and irregular intervals based on their orbit. We evaluate the performance of the method across a range of observational overpass intervals commensurate with those from operational satellites. Here, nine synthetic DA experiments are conducted where the synthetic observations, that is, *SM*_*truth*_, are assigned regular return intervals of 1 to 9 days, respectively. A 10th experiment is conducted that applies SMAP's irregular return interval to *SM*_*truth*_. Synthetic observations with SMAP's return interval are referred to as *SM*_*truth + Overpass*_.

#### 2.2.3 Observation Noise

Remotely sensed retrievals are inherently noisy, and assimilating less noisy observations is expected to result in more accurate estimates from DA simulations (Reichle et al., 2008). To this end, synthetic observations are generated by adding random 0‐mean Gaussian noise with standard errors of 0.01, 0.02, 0.03, 0.04, and 0.05 cm^3^ cm^−3^ to *SM*_*truth + Overpass*_. Random Gaussian noise settings of 0.01–0.02 m^3^ m^−3^ are optimistic, whereas reported unbiased root mean square error for state‐of‐the‐art remotely sensed measurements generally fall between 0.03 and 0.05 cm^3^ cm^−3^ (Colliander et al., 2017; Entekhabi et al., 2010; Kerr et al., 2010).

#### 2.2.4 Irrigation Magnitude

We acknowledge that irrigation water use is regionally variable depending on the amount of precipitation received and crop‐water used. We seek to evaluate performance across a range of plausible irrigation rates relative to their background precipitation, which are representative of different regions within CONUS. We analyze the method in context of seasonal precipitation magnitude over irrigated sites in Nebraska, Florida, Mississippi, California's Central Valley, and Oregon. The baseline method was evaluated in Nebraska, which has been a focus of other irrigation studies (Johnson et al., 2011; Scanlon et al., 2012; Ozdogan et al., 2010; Zaussinger et al., 2018; Jiang et al., 2014; Pun et al., 2017). These new experiments superimpose *IRRG*_*truth*_ to a range of background precipitation forcings. All truth simulations receive the same aggregated water input, for example, precipitation plus irrigation (1,380 mm per season), assuming a roughly fixed magnitude of plant water use over a single season, noting that evaporative demand in different climates will effectively increase or decrease the plant available water. A semicontinuous range of irrigation over precipitation ratios is created, ranging from 0.25 to 26.25, by rescaling precipitation and irrigation, *IRRG*_*truth*_ */P*_*Obs*_, from the *P*_*Obs*_ and *IRRG*_*truth*_ used in prior experiments with scalar multipliers. Irrigation over precipitation ratios are tested with evenly spaced intervals of 2. Experiments assimilate *SM*_*truth + Overpass*_ imposed with typical noise for the SMAP satellite, 0.03 cm^3^ cm^−3^.

#### 2.2.5 Model‐Observation Bias

The above experiments have assumed that models and observations are unbiased estimators of true SM states, specifically that the only bias present between modeled SM and observed SM is the irrigation signal. However, systematic biases between modeled SM and observed SM are widely known to exist because simulated SM is dependent upon numerous model‐specific assumptions related to soil texture, physics parameterizations, and vertical discretization (Dirmeyer et al., 2006; Koster et al., 2009). For this reason, bias correction is a common practice in DA systems, including SM DA over irrigated (Kumar et al., 2015; Nair & Indu, 2019) and nonirrigated (De Lannoy et al., 2007; Kumar et al., 2012; Reichle et al., 2004) regions. Although remotely sensed SM offers promise to improve unmodeled irrigation estimates, developing a bias correction technique that does not erase unmodeled signals such as those from irrigation, for example, in adjusting observations to the LSM climatology, remains an unresolved issue in using DA to quantify irrigation water use (Kumar et al., 2015; Nair & Indu, 2019; Zhang et al., 2018). Therefore, we evaluate the proposed method in the context of systematic biases caused by errors in modeled or observed SM, since studies like Kumar et al. (2015) suggest that this source of bias is important. While we study the role of bias on methodological performance, we do not attempt to develop a new bias correction scheme here, given the lack of consensus in bias correcting modeled and unmodeled processes.

In these experiments, a range of static systematic biases (−0.2 to 0.2 cm^3^ cm^−3^) are applied to *SM*_*truth + Overpass*_ at each time step prior to assimilation. Forty DA simulations are run, uniformly sampling the range of biases. The range of imposed biases is based on actual biases present between NLDAS‐2 LSMs and the SMAP satellite, calculated over the entire NLDAS domain (25°–53°N, 125°–67°W), excluding the top 8% most intensively irrigated regions defined in the MIRCA2000 data set (Portmann et al., 2010) and points in space‐time where SMAP was flagged for poor quality. Comparisons were made exclusively over nonirrigated or lightly irrigated regions so as to address systematic biases between LSMs and SMAP that are due to factors other than unmodeled irrigation.

#### 2.2.6 Irrigation Application Timing

While the continuous irrigation schedule applied is fairly common in the United States (Johnson et al., 2011; Ross, 1997), this scheduling is rarely applied in Europe and elsewhere. For example, in Europe a 2 days per week irrigation schedule is expected to be commonplace. We seek to evaluate DA performance when *IRRG*_*truth*_ is not applied on the previously assumed continuous schedule in scenarios where irrigation timing is both known and unknown. Unknown irrigation timing is represented by differences in timing between *IRRG*_*truth*_ and DA particles. We present results from the 24‐day window experiment in section 2.2.1 where a continuous, that is, all day every day, irrigation schedule is applied to both the truth simulation and the particles. We then conduct two experiments with new truth irrigation schedules, and these schedules are assumed to be unknown. In these experiments, particle irrigation is applied all day every day, and *IRRG*_*truth*_ is applied (i) every day only during the hours of 4 a.m.–10 a.m. (Warren & Bilderback, 2002; Park & Smith, 2008) and (ii) all day, 2 days per week (Hassanli et al., 2009; Seginer, 1967). Both of these experiments preserve the weekly and seasonal magnitude of irrigation relative to the original *IRRG*_*truth*_. Two synthetic DA experiments are conducted where *SM*_*truth*_ produced from respective truth simulations are assimilated with the ensemble of particles. To evaluate performance when irrigation schedule is known, two similar experiments are conducted where *IRRG*_*truth*_ is applied (i) every day only during the hours of 4 a.m.–10 a.m. and (ii) all day, 2 days per week, identical to the former two experiments, with the exception that in these experiments particles receive irrigation on the same schedule as *IRRG*_*truth*_, for example, assuming irrigation timing is known.

#### 2.2.7 Comprehensive Evaluation

The above experiments have introduced sources of errors on the DA system one at a time. Here we consider a combination of error sources to carry out a synthetic real‐world experiment. We run irrigation timing experiments as described in section 2.2.6, except now assimilating synthetic observations generated by adding random 0‐mean Gaussian noise with a standard error of 0.04 cm^3^ cm^−3^ to *SM*_*truth + Overpass*_. This experiment explores uncertainties of irrigation timing as discussed in section 2.2.6 in a real‐world context based on the use of realistic synthetic observations.

### 2.3 Comparison Metrics

We compute commonly used statistical performance measures between the DA system, for example, daily *IRRG*_*PBS*_, against the synthetic truth, for example, daily *IRRG*_*truth*_ exclusively during the irrigation season. These measures include percent bias (PBIAS) to help identify average biases (overprediction vs. underprediction) for irrigation estimates over an entire season, and Pearson's correlation coefficient (*R*) to quantify timing errors, or the degree of collinearity between estimated and truth irrigation (Moriasi et al., 2015).

## 3 Data Sources

### 3.1 Land Surface Model

The VIC (version 4.2.1.d; Liang et al., 1994) model is chosen for this study given its comparable complexity to other state‐of‐the‐art LSMs, its use in NLDAS‐2 (Xia et al., 2012a; Xia et al., 2018), and the Land Information System (Kumar et al., 2006; Peters‐Lidard et al., 2007). VIC is run in water and energy balance mode at an hourly time step, forced with precipitation, relative humidity, wind speed, partial vegetation cover fraction, atmospheric pressure, air temperature, incoming shortwave, and longwave radiation. All model integrations use the standard 10 cm depth for the uppermost soil layer. In real‐world applications, the depth of the upper layer should be adjusted to accommodate the sensing depth of SMAP SM (<5 cm).

### 3.2 Land Surface Model Inputs

NLDAS‐2 hourly meteorological and monthly vegetation greenness fraction data (Xia et al., 2012b) are used to force all simulations over a single grid cell in the study watershed. Relative humidity, shortwave radiation, longwave radiation, and pressure are interpolated bilinearly, while precipitation and wind are interpolated bicubically to the location of the study grid cell, given the shorter lengths of variability associated with precipitation and wind (Livneh & Hoerling, 2016). Interpolated precipitation is referred to as *P*_*Obs*_ in Table 1. Monthly NLDAS‐2 vegetation greenness fraction is uniformly disaggregated to hourly data and spatially interpolated using the nearest neighbor approach. Soil parameters were obtained from the Livneh data set (Livneh et al., 2015). For core experimentation, irrigation (819 mm per season) follows corn water use patterns in Western Nebraska (Yonts, 2002), that is, *IRRG*_*truth*_ (Table 1). For experiments exploring sensitivity to irrigation magnitude relative to precipitation, section 2.2.4, irrigation forcings follow the same pattern as that described in Yonts (2002), but scaled to seasonal magnitudes discussed in section 2.2.4.

### 3.3 SMAP

Although SMAP data are not directly used in this study, the experimental set up is guided by key SMAP attributes, for example, overpass time, frequency, and shallow sensing depth. SMAP provides morning and evening (6 a.m. and 6 p.m. local time) estimates of surface SM, globally every 1–3 days (Entekhabi et al., 2014), has a sensing depth of approximately 0–50 mm, and meets the mission goal of 0.04 mm^3^ mm^−3^ unbiased root mean square error (Chan et al., 2018; Colliander et al., 2017). Here, we consider only the 6 a.m. overpasses because the SMAP algorithm assigns a single temperature to both the soil and its overlying canopy, a condition that is best met in the morning hours (Entekhabi et al., 2014; Jackson et al., 2012). We exclude SMAP data that have been flagged for uncertain quality.

## 4 Results

Figure 2 illustrates the performance of the key elements of the DA system, the translation of assimilation weights from surface SM into the single best estimate irrigation over a growing season. The color of the 99 lines in Figure 2a shows the weights assigned to each particle in the PBS algorithm, assigned based on their proximity to synthetic observations, shown in red dots, in each fixed window. The vertical gray dashed lines denote 16‐day fixed window bounds. A weighted average of the particles is considered the best estimate SM time series (black line in Figure 2a). This best estimate closely matches *SM*_*truth*_ and therefore accurately reflects the effects of truth irrigation on modeled SM, unlike OL SM that lacks knowledge of irrigation (dashed back line). For clarity, Figure 2a only shows a portion of the irrigation season, but the same approach is applied to the entire time period shown in Figure 2b.

The translation of particle weights from SM DA into precipitation and irrigation weights is shown in Figure 2b, producing an estimated irrigation time series, *IRRG*_*PBS*_ (black line with gray band of uncertainty in Figure 2b). The results from this baseline experiment, prior to introducing errors into the daily synthetic observations, yield PBIAS and *R* values of 0.66% and 0.95, respectively. The purpose of this study is to assess how well the PBS (*IRRG*_*PBS*_) can be used to estimate *IRRG*_*truth*_ (Figure 2b, red line) when considering the variety of errors that are likely to be present when assimilating remotely sensed SM.

### 4.1 Window Length

**Figure 3:**
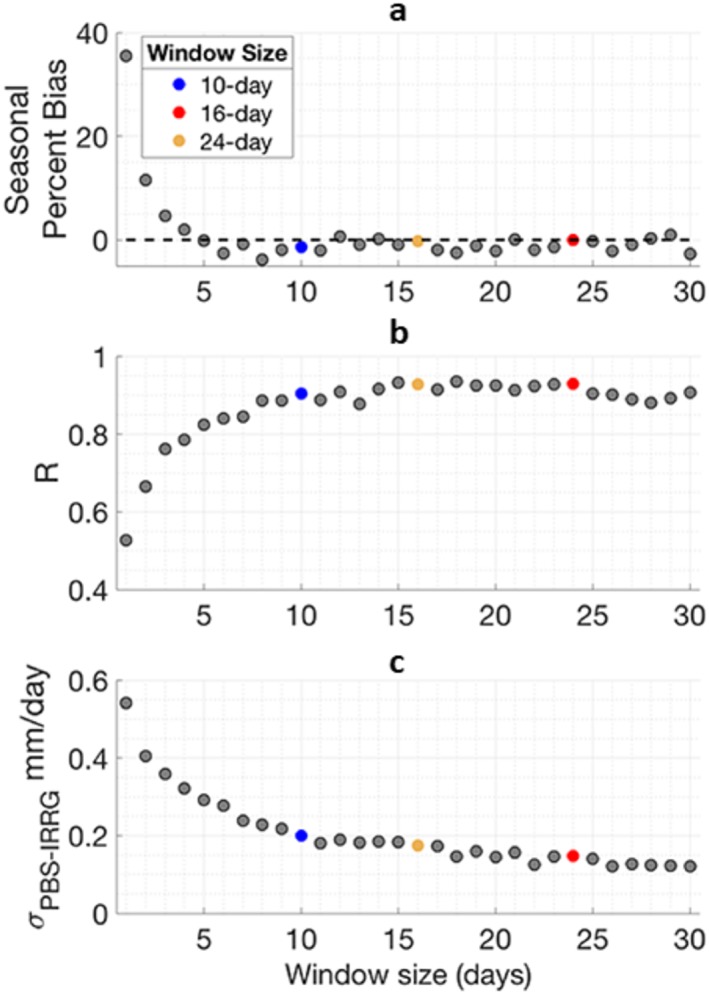
Performance of estimated irrigation produced from data assimilation experiments assimilating *SM*_*truth*_ using window lengths of 1–30 days. Dots represent median summary statistics for each window length from a suite of 10 synthetic data assimilation experiments initialized from staggered start dates. (a) Absolute PBIAS comparing *IRRG*_*PBS*_ with *IRRG*_*truth*_. (b) *R* comparing *IRRG*_*PBS*_ with *IRRG*_*truth*_. (c) Uncertainty of *IRRG*_*PBS*_ (*σ*_*IRRG* − *PBS*_).

The performance of irrigation estimates improves with increasing window length for all skill metrics until it plateaus at a window length longer than approximately 10 days, beyond which (10‐ to 30‐day window lengths) PBIAS is less than 2%, *R* is greater than 0.9, and the uncertainty, *σ*_*IRRG* − *PBS*_, is less than 0.2 mm day^−1^ (see Figure 3). When window lengths are short (<10 days), errors between particle SM and observed SM tend be dominated by the initial states of the fixed window rather than particle forcing. Longer window lengths shrink the effect of particles' initial condition on PBS assigned weights and allows for weights to be driven by the accuracy of model inputs (e.g., irrigation). The particle filter is considered a special case of the PBS when the window length is 1 day. Hence, a PBS is more effective for estimating irrigation than a particle filter. In a multiobjective optimization context, the 16‐ and 24‐day windows can be considered roughly equal performing, or nondominated relative to each other, and the 10‐day window is dominated, or outperformed, by both the 16‐ and 24‐day windows.

*σ*_*IRRG* − *PBS*_ is related to the spread of weights assigned in the particle smoothing algorithm. A small *σ*_*IRRG* − *PBS*_ indicates that there is a narrow grouping of particles receiving high weights with the extreme case being that only one particle receives weight. This extreme case is approached either as observational uncertainty, *C*_*v*_, from equation (3), decreases or as the ensemble variance increases. Longer windows allow for the spread of the ensemble to expand, thus explaining the narrowing *σ*_*IRRG* − *PBS*_ with window length. Here, decreasing *σ*_*IRRG* − *PBS*_ also corresponds with degeneracy in the PBS algorithm. For cases of the 10‐, 16‐, and 24‐day windows, the mean number of particles resampled during the irrigation season in the sequential importance resampling process are approximately 22, 18, and 15, respectively. This supports that degeneracy is more pronounced with an increasing window length.

### 4.2 Frequency of Observations

**Figure 4:**
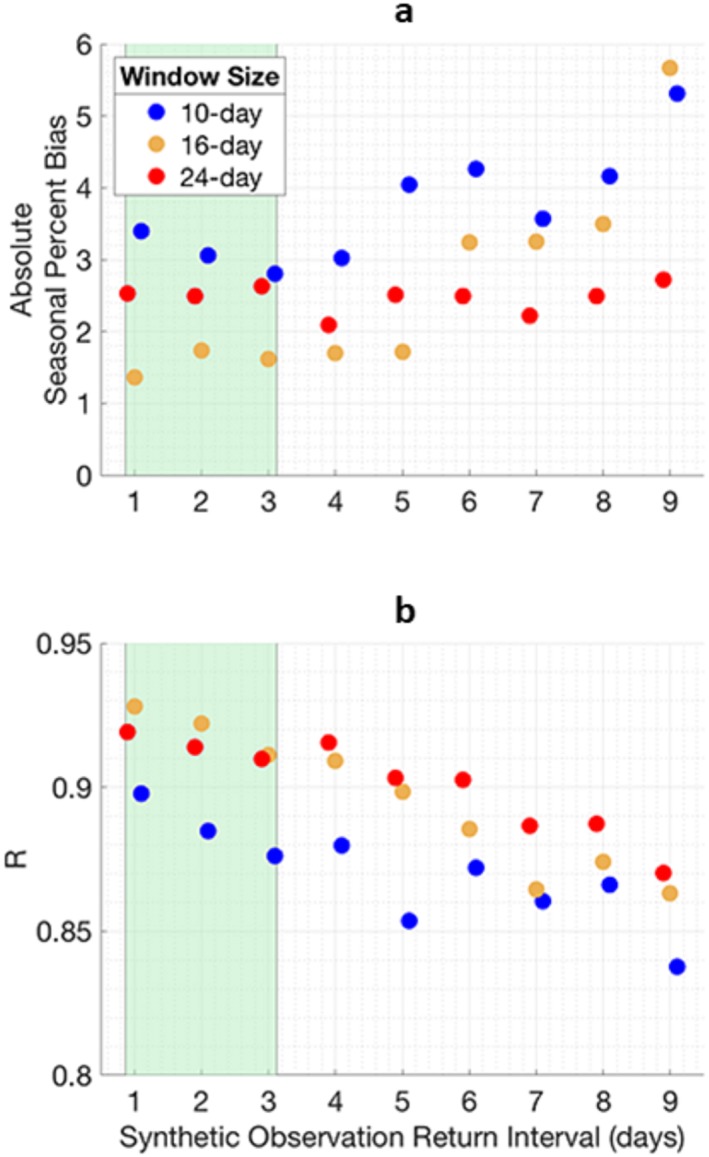
Performance of estimated irrigation produced from data assimilation experiments assimilating *SM*_*truth*_ with regular return intervals shown on the horizontal axis. The green highlighted region represents return interval range of SMAP. Dots represent median values from 50 synthetic data assimilation experiments for each return interval scenario. (a) Absolute PBIAS comparing *IRRG*_*PBS*_ with *IRRG*_*truth*_. (b) *R* comparing *IRRG*_*PBS*_ with *IRRG*_*truth*_.

More frequent observations yield more accurate estimates of irrigation, with longer window lengths more robust to the variability of infrequent observations as shown in Figure 4. Longer windows tend to be more stable because more observations are assimilated within each fixed window relative to shorter window lengths. A key threshold is seen in the 10‐day window case (blue dots), for return intervals of 5 days or greater since two or fewer observations can be accounted for in each fixed window. When too few observations are assimilated in a fixed window, the irrigation signal can be overwhelmed by noise in precipitation forcing, and errors are more likely to be propagated forward from one window to the next by resampling particles from inaccurate initial conditions. The green highlighted region in Figure 4 represents the range of return interval of the SMAP satellite (Entekhabi et al., 2014). Here, all tested windows produce a median absolute PBIAS of less than 3.4%, 16‐ and 24‐day windows yield a median *R* of at least 0.91, with the simulations using a 10‐day window yield a median *R* of at least 0.88. *σ*_*IRRG* − *PBS*_ (not shown) is effectively insensitive to return interval.

We also conducted a similar experiment to Figure 4 where we imposed irregular return intervals such as those from SMAP, assimilating *SM*_*truth + Overpass*_ as synthetic observations (not shown) yielding a median PBIAS of less than 1% and median *R* of greater than 0.90 for simulations using 16‐ and 24‐day windows. Simulations using a 10‐day window yield slightly degraded performance with a median PBIAS of 2.57% and median *R* of 0.87. Results from experiments using irregular overpass intervals are consistent with performance from experiments using regular overpass intervals, indicating that the return intervals from SMAP are generally not a major limiting factor to overall performance.

### 4.3 Observation Noise

**Figure 5:**
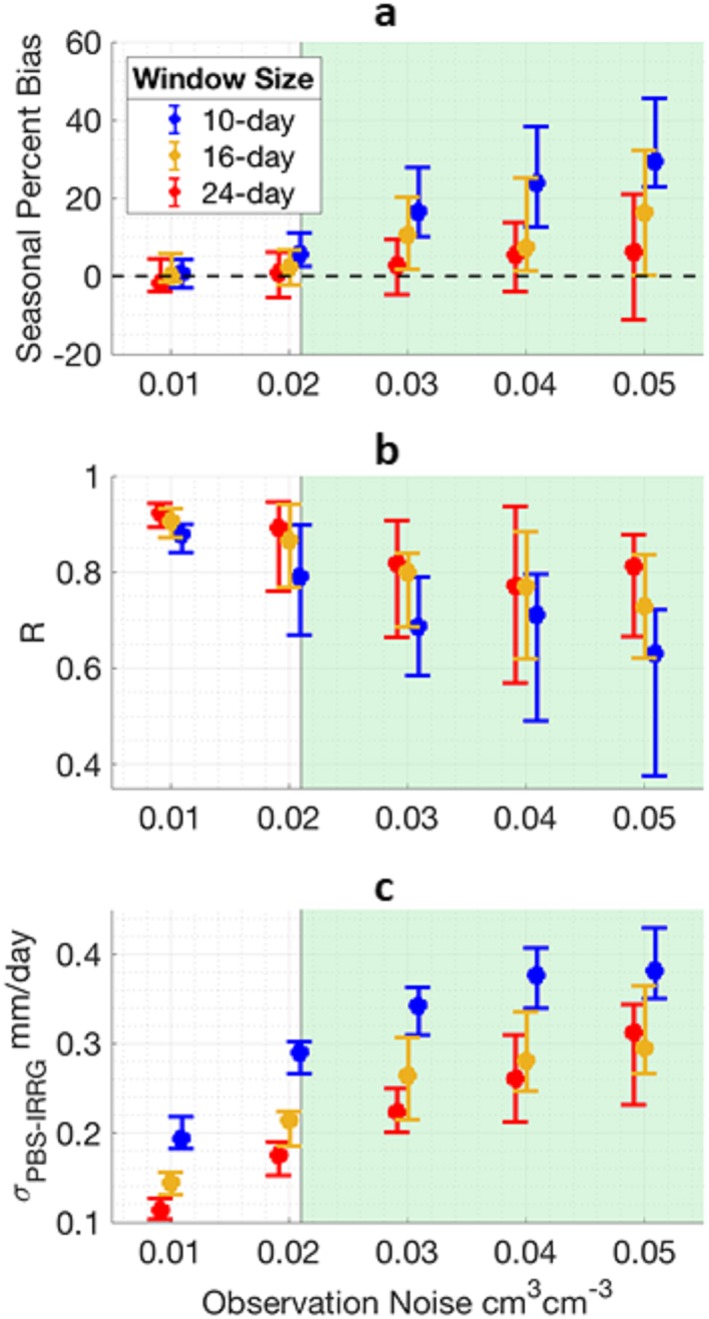
Performance of estimated irrigation produced from DA experiments assimilating *SM*_*truth + Overpass*_ perturbed with 0‐mean Gaussian noise with a standard error denoted by the horizontal axis. Twenty simulations are run for each observational noise scenario, producing a new time series of perturbed observations each simulation. Filled circles represent the median summary statistic from the 20 simulations, and upper and lower error bars represent the 85th and 15th percentiles. The green highlighted region represents the reported range of unbiased noise from SMAP at core validation sites. (a) Absolute PBIAS comparing *IRRG*_*PBS*_ with *IRRG*_*truth*_. (b) *R* comparing *IRRG*_*PBS*_ with *IRRG*_*truth*_. (c) Uncertainty of *IRRG*_*PBS*_ (*σ*_*IRRG* − *PBS*_).

DA experiments with more noisy observations yield both less accurate and more uncertain estimates of irrigation, with the performance of longer windows less impacted by noise. Figure 5 shows the median performance and its uncertainty and highlights the range of error standard deviations (0.021–0.056 cm^3^ cm^−3^) expected from SMAP's mission (Colliander et al., 2017). For experiments imposing noise equivalent to SMAP's mission goal (0.04 cm^3^ cm^−3^), experiments using a 10‐day window show a median seasonal PBIAS and correlation of 16% and 0.54, respectively. Experiments using a 16‐day window show a median PBIAS and *R* of 10% and 0.74, respectively. Experiments using a 24‐day window show the best performance with a median PBIAS and *R* of 1% and 0.76, respectively. Figure 5c corroborates Figure 3: Uncertainty, *σ*_*IRRG* − *PBS*_, is generally lower for longer windows. Figure 5c also shows that *σ*_*IRRG* − *PBS*_ increases with observational noise, which is a direct reflection of the PBS likelihood formulation (equation (3)) that drives particle weighting. As the error covariance of the synthetic observations increase, particle weighting becomes more uniform, that is, more particles receive relatively high weights, in turn the uncertainty (equation (4)) increases.

### 4.4 Irrigation Magnitude

**Figure 6:**
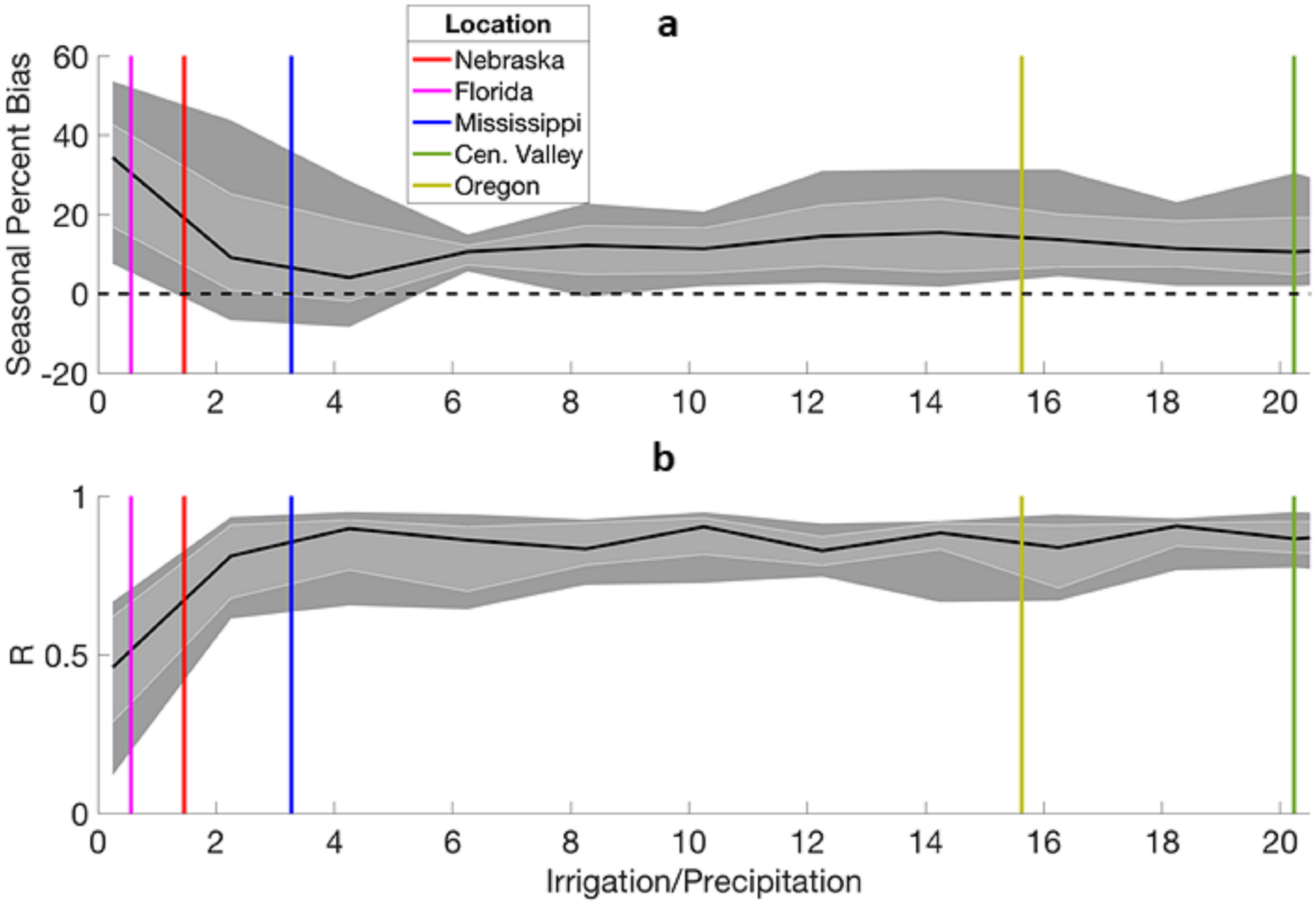
Performance of estimated irrigation across a range of seasonal irrigation versus precipitation ratios. Dark gray bands represent the 10th and 90th percentiles, and light gray represents the 25th and 75th percentiles of summary statistics from the 20 synthetic data assimilation experiments for each tested irrigation/precipitation ratio. The black line represents the median summary statistic from the 20 data assimilation experiments with vertical colored lines reflecting estimated irrigation over precipitation ratios for five sites (site locations displayed in Figure 7).

The DA system shows increasing skill as the ratio of irrigation/precipitation (I/P) increases until the ratio reaches approximately 4, with known irrigation regions within CONUS shown by vertical colored lines in Figure 6. Experiments where irrigation is small compared to precipitation (I/P < 4) produce larger errors because multiplicative precipitation perturbations and noise in the synthetic SM observations are large compared to the irrigation signal. The persistent positive PBIAS is reflective of the skewness of the 99‐member ensemble toward the uniform particle irrigation estimates, for example, 0–30 mm day^−1^, which are larger than the amount of *IRRG*_*truth*_, for example, roughly 2.6 mm day^−1^ for smaller I/P ratios toward the left part of Figure 6. In this case, the maximum underestimation for irrigation is approximately 2.6 mm day^−1^, whereas the maximum overestimation of irrigation is approximately 27.4 mm day^−1^. Therefore, random noise in synthetic SM observations and precipitation perturbations tends to favor overestimates of irrigation. It is worth noting that this artifact would be removed if the range of irrigation applied to the particles were perfectly symmetric about *IRRG*_*truth*_. Overall, it appears that the DA system tends to produce positive biases across both drier and wetter climate regimes where irrigation plays larger or smaller roles, and application of this method over wetter climates where irrigation plays smaller roles will produce less skilled estimates of irrigation.

### 4.5 Systematic Bias

**Figure 7:**
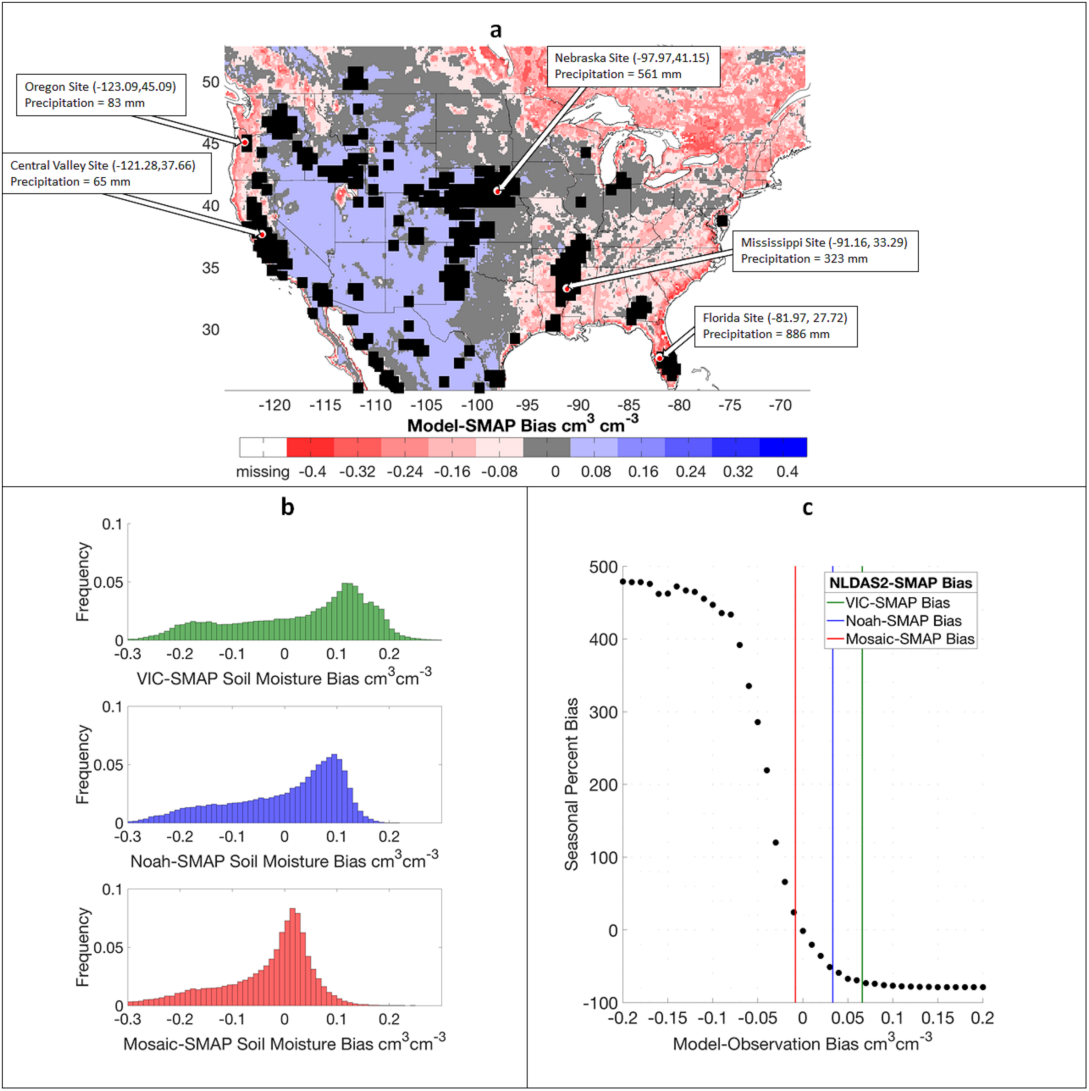
(a) Biases between NLDAS‐2 ensemble (VIC, Noah, and Mosaic) mean surface SM and SMAP surface SM. Irrigated locations excluded from the bias histogram in (b) are shown as black boxes. Latitude, longitude, and respective precipitation amounts during the irrigation season for sites discussed in sections 2.2.4 and 4.4 are included. (b) Histograms of mean biases between three NLDAS‐2 LSMs and SMAP over nonirrigated regions. (c) PBIAS comparing *IRRG*_*PBS*_ with *IRRG*_*truth*_. *IRRG*_*PBS*_ is estimated from data assimilation experiments assimilating *SM*_*truth + Overpass*_ perturbed with a temporally static bias (model‐observation biases shown on the horizontal axis). Vertical colored lines represent the median error‐based bias between respective LSMs and SMAP, derived from the histograms in (b).

Biases between NLDAS‐2 LSMs and SMAP are computed across the CONUS, Figures 7a and 7b, revealing that systematic LSM‐SMAP biases are often large enough to potentially dominate DA performance, noting that we assume the LSM 10 cm depth for the uppermost soil layer is comparable to the SMAP sensing depth of <5 cm. When these systematic biases exceed ±0.01 cm^3^ cm^−3^, seasonal PBIAS performance exceeds 20%. Figure 7c shows the resulting PBIAS of the DA system when subjected to biases imposed to *SM*_*truth + Overpass*_ prior to assimilation. In experiments where model‐observation biases are positive, relatively dry observations favor particles forced with lower irrigation quantities resulting in underestimations of irrigation. In experiments where model‐observation biases are negative, relatively wet observations favor particles forced with higher irrigation quantities resulting in overestimations of irrigation. Results indicate <20% seasonal PBIAS can be obtained from only 3.6%, 5.0%, and 13.6% (for VIC, Noah, and Mosaic, respectively) of locations without bias correction, underscoring the importance of systematic bias and identifying a suitable correction scheme. Biases between models and observations present a large obstacle for the usability of the method until advances are made in SM bias correction that preserve observed irrigation signal or models are successfully calibrated to produce unbiased SM estimates relative to observations.

### 4.6 Irrigation Application Timing

**Figure 8:**
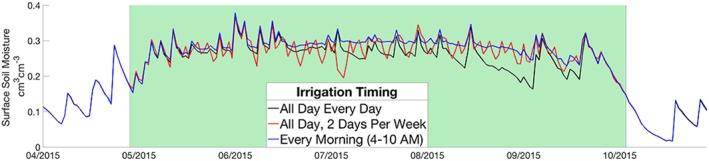
Three *SM*_*truth*_ time series from the same amount of irrigation applied: all day, every day (black), every morning (blue), and all day two times per week (red). The irrigated season is highlighted in green.

Discrepancies between assumed and actual irrigation timing can result in important errors between *IRRG*_*PBS*_ and *IRRG*_*truth*_. Irrigation timing sensitivities are similar in magnitude and characteristic to model‐observation bias sensitivities described in the previous section. When *IRRG*_*truth*_ timing produces wetter states than *IRRG*_*PBS*_ timing (for the same magnitude of irrigation), the LSM will have a dry bias relative to *SM*_*truth*_. For example, evaporative losses are reduced in a scenario where irrigation is applied only during morning hours, thus resulting in wetter states than cases where irrigation is applied throughout a day. Also, similar to Haddeland et al. (2002), a simulation with uniform irrigation application, that is, all day every day, results in drier states than the same amount of irrigation applied in higher concentrations, that is, 2 days per week. Figure 8 shows the SM time series for the three truth simulations that apply irrigation on different schedules (explained in section 2.3.6). Table 3 reports the mean SM over the irrigated season for each truth simulation, and the resulting summary statistics from five DA experiments described by the timing of *IRRG*_*truth*_ and particle irrigation. As expected, the experiment that applied *IRRG*_*truth*_ each morning yielded a relatively wet *SM*_*truth*_ compared to particles that applied irrigation continuously each day, resulting in *IRRG*_*PBS*_ with the largest PBIAS (56%). However, *IRRG*_*PBS*_ from this experiment captured the temporal variation of irrigation well, indicated by a high *R* (0.91). When assumed irrigation timing matches the timing of *IRRG*_*truth*_, performance on a weekly time step is consistent for the three tested irrigation schedules (see gray rows in Table 3). These results indicate that irrigation timing is not a limiting factor to the application of this method on a weekly time scale. However, if irrigation timing is unknown *IRRG*_*PBS*_ will exhibit large biases relative to *IRRG*_*truth*_, particularly for shorter time scales.

**Table 3:** Summary Statistics From the Five Time Sensitivity Experiments, Where the Mean Irrigation Season Soil Moisture Represents the Mean Soil Moisture From Respective Truth Simulations Over the Irrigated Season and Irrigation Summary Statistics Are Calculated Comparing *IRRG*_*PBS*_ With *IRRG*_*truth*_ From the Five DA Experiments

True irrigation schedule	Particle irrigation schedule	Mean irrigation season moisture (cm^3^ cm^−3^)	Irrigation summary statistics	
			PBIAS (%)	*R* daily (weekly)
All day, every day	All day, every day	0.26	−1	0.94 (0.95)
Every morning (4–10 a.m.)	All day, every day	0.28	53	0.91 (0.95)
Every morning (4–10 a.m.)	Every morning (4–10 a.m.)	0.28	4	0.94 (0.97)
All day, 2 days per week	All day, every day	0.27	28	0.11 (0.94)
All day, 2 days per week	All day, 2 days per week	0.27	1	0.79 (0.97)

*Note*. Rows shaded in gray indicate experiments where *IRRG*_*truth*_ and particle irrigation are applied on identical schedules, representing “known” irrigation timing.

Since this method estimates the amount of irrigation needed to achieve target SM states for a given set of inputs, including assumed irrigation timing, these types of experiments could conceivably be used to determine the efficiency of an irrigation schedule. For example, a suite of alternative irrigation schedules could be evaluated to identify the most efficient approach to achieve a target SM for a given crop.

### 4.7 Comprehensive Evaluation

Observational noise and an irregular return interval only slightly reduces the correlation between *IRRG*_*PBS*_ and *IRRG*_*truth*_ as seen when comparing the median from synthetic experiments that impose noise on *SM*_*truth + Overpass*_ (Table 4) with experiments that use perfect observations (Table 3). In all cases, *IRRG*_*PBS*_ has a higher correlation with *IRRG*_*truth*_ on a weekly, rather than daily, time step. This is especially true when *IRRG*_*truth*_ is applied 2 days per week, but the assumed irrigation schedule is continuous. *IRRG*_*PBS*_ produced from experiments assuming known timing of *IRRG*_*truth*_ are nondominated in a multiobjective optimization context relative to each other (see gray rows in Table 4). Hence, this method is not timing specific and can be used over a range of irrigation schedules, but the accuracy is heavily dependent on a priori knowledge of irrigation timing. Errors in *IRRG*_*PBS*_ are dominated by issues that arise from discrepancies in timing between particle irrigation and *IRRG*_*truth*_ rather than observational noise or frequency for experiments assuming the timing of *IRRG*_*truth*_ is unknown (see white rows in Table 4).

**Table 4:** Summary Statistics From the Five Time Sensitivity Experiments, Where Irrigation Summary Statistics Are Calculated Comparing *IRRG*_*PBS*_ With *IRRG*_*truth*_ From the Five DA Experiments

True irrigation schedule	Particle irrigation schedule	Irrigation summary statistics		
		PBIAS (%)	Daily *R*	Weekly *R*
		PCTL (15, 50, 85)	PCTL (15, 50, 85)	PCTL (15, 50, 85)
All day, every day	All day, every day	(−10, 0, 13)	(0.59, 0.69, 0.84)	(0.83, 0.88, 0.94)
Every morning (4–10 a.m.)	All day, every day	(34, 61, 95)	(0.62, 0.75, 0.85)	(0.86, 0.91, 0.93)
Every morning (4–10 a.m.)	Every morning (4–10 a.m.)	(−2, 13, 26)	(0.68, 0.80, 0.86)	(0.87, 0.93, 0.95)
All day, 2 days per week	All day, every day	(15, 34, 60)	(0.05, 0.07, 0.08)	(0.87, 0.91, 0.93)
All day, 2 days per week	All day, 2 days per week	(−25, −5, 4)	(0.72, 0.75, 0.79)	(0.78, 0.86, 0.94)

*Note*. Rows shaded in gray indicate experiments where truth irrigation timing is assumed to be known. The 15th, 50th, and 85th percentiles are reported for each statistic from the 20 simulations conducted for each timing scenario.

## 5 Discussion and Conclusions

In this study, we evaluate a new approach for estimating irrigation magnitude by assimilating SM with an LSM. Through synthetic experiments, the sensitivity of the DA system is assessed relative to (i) the window length of the PBS algorithm, (ii) the frequency of observations, (iii) the amount of noise in the SM data, (iv) the relative magnitude of irrigation compared to precipitation, (v) the magnitude of biases between models and observations, and (vi) the timing of irrigation. Experiments are designed in the context of assimilating observations from the SMAP satellite with the VIC LSM. However, this DA system can be used with a wide variety of SM observations and models. Based on the above results, the following conclusions can be drawn:

1. DA experiments using synthetic observations assigned SMAP's overpass schedule and zero random noise produce accurate irrigation estimates (PBIAS < 2% and *R* > 0.9) for window lengths longer than 10 days. Therefore, smoothing DA algorithms, for example, the PBS, must be used with this method rather than filtering DA algorithms, for example, the particle filter, that assimilate single data points in isolation. Experiments using a large window length, for example, 24 days, are more robust to overpass frequency and observation noise because longer window lengths assimilate a greater number of observations in each window. This provides the algorithm with more irrigation signal relative to random observational noise and increases the likelihood of better irrigation estimates. These results are also consistent with Brocca et al. (2018) where irrigation data were aggregated at a monthly time scale to reduce the influence of observation noise.
2. DA performance is strongly related with the frequency of observations, where more frequent return periods (e.g., every 2–3 days) produce more accurate estimates of irrigation than experiments using synthetic observations with less frequent return periods (e.g., weekly or longer). Moving from synthetic observations with regular intervals to SMAP's irregular return period does not appreciably hinder performance.
3. Performance is directly affected by the amount of random noise in the signal. Experiments using synthetic observations perturbed with less random noise (0.01–0.02 cm^3^ cm^−3^) yield better performance than experiments using synthetic observations perturbed with larger random noise (0.03–0.04 cm^3^ cm^−3^). Experiments that assimilate synthetic observations with both SMAP's return period and expected observational noise (0.04 cm^3^ cm^−3^) produce estimates of irrigation with a median PBIAS of 1% and *R* of 0.76, and range of PBIAS and *R* of −3.98% to 13.85% and 0.57–0.94, respectively.
4. The presented methodology is likely to overestimate irrigation when the magnitude of true irrigation is small in comparison to the range of particle irrigation because multiplicative precipitation perturbations dominates the irrigation signal in these cases. Further, when constraints on *IRRG*_*PBS*_, that is, range of irrigation applied to particles, are asymmetric about *IRRG*_*truth*_, *IRRG*_*PBS*_ will be underestimated or overestimated based on the direction of the skewness. In this study the upper constraint (30 mm day^−1^) was further from *IRRG*_*truth*_ (3–8 mm day^−1^) than the lower constraint (0 mm day^−1^), thus causing random noise in synthetic SM observations to favor overestimated irrigation throughout a season.
5. A large obstacle to implementing this method is the systematic bias between LSMs and observations. Bias correction is necessary to implement this method to produce reliable real‐world irrigation estimates over large areas. This analysis underscores the importance of developing and testing new bias correction methods that will not erase unmodeled processes like irrigation.
6. The largest obstacle to implement this method is a priori knowledge of irrigation timing. When irrigation timing is assumed to be known, the method is able to accurately predict the magnitude and temporal pattern of *IRRG*_*truth*_ for a suite of irrigation schedules, but large errors arise in experiments where the timing of *IRRG*_*truth*_ is assumed to be unknown. The presented algorithm estimates irrigation magnitudes given model inputs, including an assumed irrigation scheduling. Therefore, the method is partially limited by knowledge of the true irrigation schedule such that discrepancies in schedule between particulate and truth irrigation can result in systematic biases between observed and modeled SM. These SM biases propagate to biases in irrigation estimates. Although this presents a limitation to the method, it also provides the opportunity to use the method to assess the efficiency of irrigation strategies as a function of model inputs.

This study presents an evaluation of a new method to estimate irrigation quantities using the PBS DA method. Future studies that seek to evaluate the method in nonsynthetic applications should first address the issue of identifying irrigation timing and also explore ways to correct biases between modeled and observed SM so as to preserve the natively observed irrigation signal. Because bias correction is an active area of research it may be easier to resolve than knowledge of irregular irrigation timing. Both of these are valuable pre‐processing steps to a successful application of this methodology. Applications of this method will require identification of the start and end dates of the irrigation season at the model's spatial resolution by employing methods such as that presented in Lawston et al. (2017) that showed comparing SMAP SM to in situ precipitation data can identify the seasonal onset of irrigation. Future efforts to advance the science of irrigation estimation through DA could assess a priori bias correction methods in a bias aware DA system and build upon ongoing LSM SM calibration efforts, for example, by NASA's Land Information System team, or assess CDF‐matching observations to the climatology of an LSM using an irrigation scheme, or could explore assimilating multiple remotely sensed variables that contain irrigation signal. Alternatively, it may be fruitful to assess posterior bias correction strategies such as removing bias from estimated irrigation by estimating model bias over adjacent nonirrigated cropland areas and use these to correct simulations over irrigated pixels (Jalilvand et al., 2019). The issue of bias may also be ameliorated by using a different type of model, for example, Hydrus‐1D, which has been shown to agree relatively well with SMAP retrievals at core validation sites (Small et al., 2018).
